# Diet Shift and Its Impact on Foraging Behavior of Siberian Crane (*Grus Leucogeranus*) in Poyang Lake

**DOI:** 10.1371/journal.pone.0065843

**Published:** 2013-06-18

**Authors:** Yifei Jia, Shengwu Jiao, Yamian Zhang, Yan Zhou, Guangchun Lei, Guanhua Liu

**Affiliations:** 1 Beijing Forestry University, Beijing, China; 2 Jiangxi Poyang Lake National Natural Reserve Authority, Nanchang, Jiangxi Province, China; University of Sydney, Australia

## Abstract

The study of habitat selection and diet has a long history in ecology. This is often used to assess the functional roles of wetland in biodiversity conservation. Shifting habitat and diet may be one of the survival strategies during extremely adverse conditions. Therefore, sudden changes in habitat selection may indicate the deterioration of the habitat quality, and management interventions are necessary. Siberian crane (*Grus leucogeranus*) became critically endangered due to loss of habitat, and is currently a global conservation focus. Every winter, more than 95% of the species' global population congregates at Poyang Lake, and feeds on tubers of *Vallisneria spiralis* in shallow water and mudflat habitat. In this study, we reported the first sighting of large numbers of Siberian cranes foraging at wet meadows, where they fed on a different plant, *Potentilla limprichtii* due to extreme scarcity of their preferred tuber. To understand how well the cranes adapted to such unusual habitat, field surveys to assess the distribution of cranes across different habitats, and food availability in each habitat were carried out in the winter of 2011. Field observations on crane behaviors at different habitats were also conducted. Results show that cranes displayed significantly different behavior patterns when using the wet meadow, compared to the crane's optimal habitat - shallow water and mudflat. Both juveniles and adults spent significantly less time foraging, and more time alerting in meadows than in shallow waters and mudflats. These results indicated that the meadow might be a suboptimal wintering ground for Siberian crane, which helped the cranes survive from extreme unfavorable conditions. To some degree, this finding alleviates the general concern over the fluctuating of its food resources which was caused by hydrological disturbances. However, more studies are needed to assess the consequences of such diet and habitat shift for crane survival.

## Introduction

The implications of major habitat shifts for endangered species management maybe emphasis that these shifts can be negative (they imply a problem as a cause of the shift, for example, deterioration of the habitat quality [1]), but also positive (they suggest that that a species has behavioral flexibility, a strategy for surviving extremely adverse conditions [2], [3]). The change of foraging habitat usually follows the shift of diet. In Dashanbao National Nature Reserve, Black-necked cranes (*G. nigricollis*) change their home range frequently, in which food sources were the primary determinant factor [4].The forager's diets usually follow the optimal foraging theory [5], [6], [7]. And each forager's diet also adapts its own morphology. However the diet of a forager is not unchallengeable. There are many situations causing diet shifts [8]. Recolonization results in diet shift in wolverine (*Gulo gulo*), a facultative scavenger, and the wolves tend to expand their recipe [9]. However, other foragers tend to have specialized recipes for higher feeding efficiency. And their morphology is usually highly adapted with particular food.

The Siberian crane (*G. leucogeranus*) is one of the critically endangered avian species in the world [10]. There are three Siberian crane sub-populations, which all breed in the tundra of northern Russia [11]. After fledging, they migrate thousands of kilometers to the wintering grounds in Iran, India and China [12]. Populations in India and Iran have declined to less than ten individuals due to habitat loss and hunting [13]. The current size of the eastern population, which winters in China is estimated to be 3,500–3,800 [14], therefore the wintering ground in China holds the greatest potential for the welfare of the species. At the end of 2006, Barter et al. [15] recorded 2,760 individuals or 93% of the estimated Eastern population in the Poyang Lake region. In 2011, our annual bird census recorded 2,495. These demonstrated the importance of Poyang Lake for the Siberian crane. However, environmental conditions of the Lake are rapidly changing with the recent economic development, in particular, with hydro-engineering projects. A number of problems have been identified potentially threatening the ecological integrity of the Lake, including increased nutrients and sediment loading [16], reduced lake size [17], and altered inundation regimes due to the operation of the Three Gorges Dam [18]. These issues are of great concern for the conservation of the Siberian crane and other species including the endangered oriental stork (*Ciconia boyciana* Swinhoe) [15].

Siberian crane's distinctive morphology, vocalization, and feeding and courtship behavior distinguish itself from other crane species [19]. It also has special habitat requirements, exclusively using wetlands for nesting, feeding, and roosting [12]. Siberian cranes are most frequently observed wading and probing for food (tubers of submerged plants, *V. spiralis*), in shallow (up to 30 cm deep) waters [12]. The long bill and toes, and long serrated beak of Siberian craneare highly adapted the shallow water environment specialized for probing tubers. According to optimal foraging theory [5], [6], [7], abundant *V. spiralis* that grows in shallow water area where is far away from human disturbance, could be the best choice for Siberian crane. However, we observed cranes foraging on wet *Carex* meadow during our routine bird survey in the Poyang Lake National Natural Reserve (PLNNR) in January 2011. During the following monitoring, we found more Siberian cranes landing and feeding at these grassy meadows. By combining food survey and behavior observation, we try to find answers of the two questions, which are fundamental for the conservation of the endangered species in changing environment: why did Siberian cranes abandon its preferred food and habitat? How well did the cranes adapt to the new habitat?

Behavioral study has a long history in ecology and evolution [20], and is the key to understand the life history strategy of a species [21]. Lewis found *Pieris rapae* have to learn how to forage on a special flower with high efficiency [22]. *Acomys cabirinus* evolved to responses to naturally occurring odours [23]. In crane behavioral studies, researchers have been always focusing on time-budget [24], foraging behavior [25] and vigilance behavior [26]. To our best knowledge, there is few published literature studying the behavior time-budget of Siberian crane in Poyang Lake. In order to understand the fundamentals underlying the observed diet and habitat shift, we carried out field survey on the spatial distribution of Siberian cranes, and on food plants at their feeding sites. We also conducted detailed time-budgeting for the behaviors of Siberian crane in three alternative habitats, namely wet meadow, mudflat and shallow water to test the optimal foraging theory. We hypothesize that: 1) food scarcity drove Siberian crane to wet meadows; 2) the overall behavior patterns would be different in alternative habitats; and 3) the Siberian cranes would spend more time in alert behaviors in the less safe wet meadow habitat.

## Methods

This study was authorized by the Poyang Lake National Nature Reserve Authority.

### The study site

**Figure 1:**
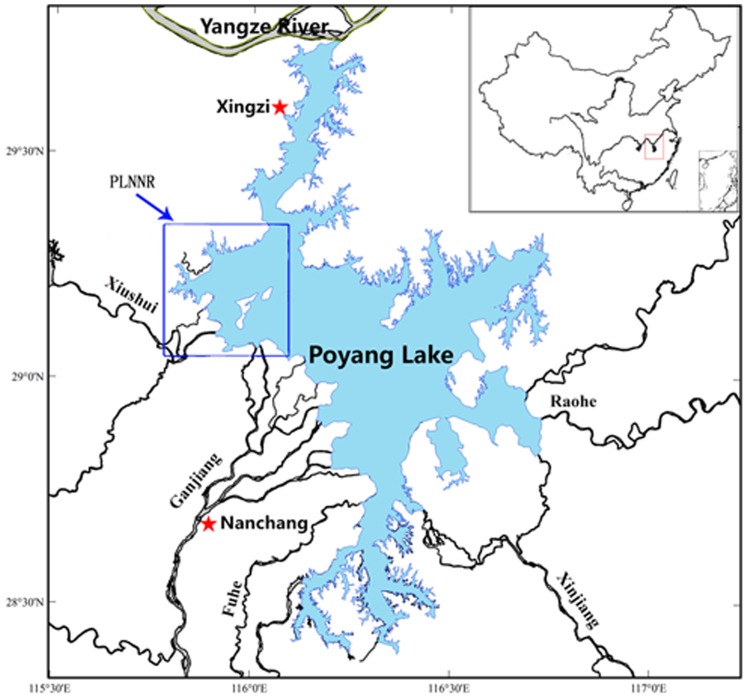
Map of Poyang lake (light blue colored area) and its five major tributaries.

**Figure 2:**
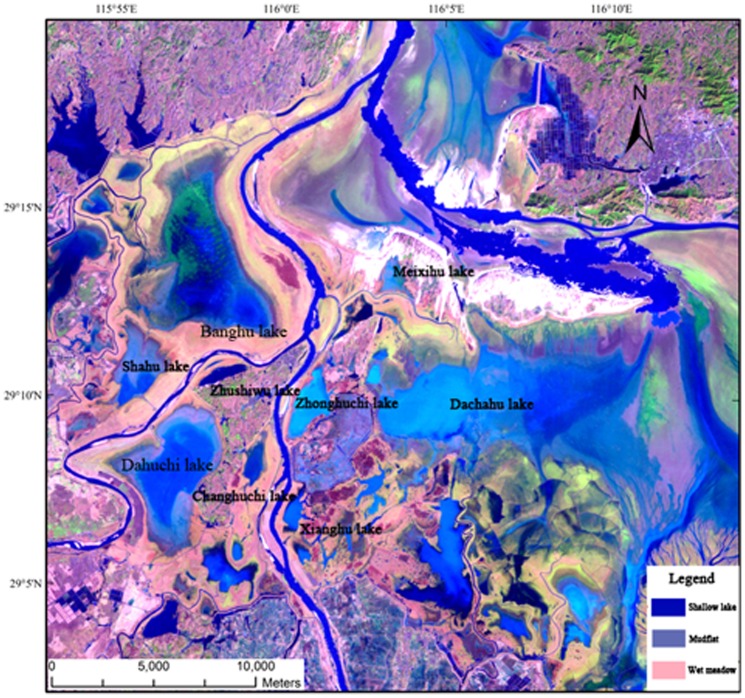
Map of Poyang Lake National Natural Reserve in winter season. The blue colored area shows shallow water habitat, light blue belt around the shallow water area is mudflats and red area is wet meadow.

Poyang Lake (28°22′–29°45′N, 115°47′–116°45′E, Figure 1) is one of the largest freshwater wetland complexes in China with an area of approximately 4,000 km^2^ in high water season [27]. Its inundation regimes and water level fluctuations are largely controlled by the dynamic balance of the five major tributaries (the Ganjiang River, the Fuhe River, the Xinjiang River, the Raohe River and the Xiushui River, Figure 1) and the Yangtze River, which in turn influenced by the subtropical monsoonal climate. During the low-water winter months (November–March), the Lake system becomes a complex assembly of hydrological distinct rivers and shallow waters interspersed with meadows (Figure 2). Such spatial heterogeneity provides diverse habitats for a range of biota, especially waterfowls. It is also during the low-water winter period when migratory waterbirds use the habitats. Over 400,000 migratory waterbirds use the wetlands as wintering ground each year [15], [28], which include 95% of the global Siberian cranes population, 57% of the global oriental white storks population, and other 17 endangered species [29].

**Figure 3:**
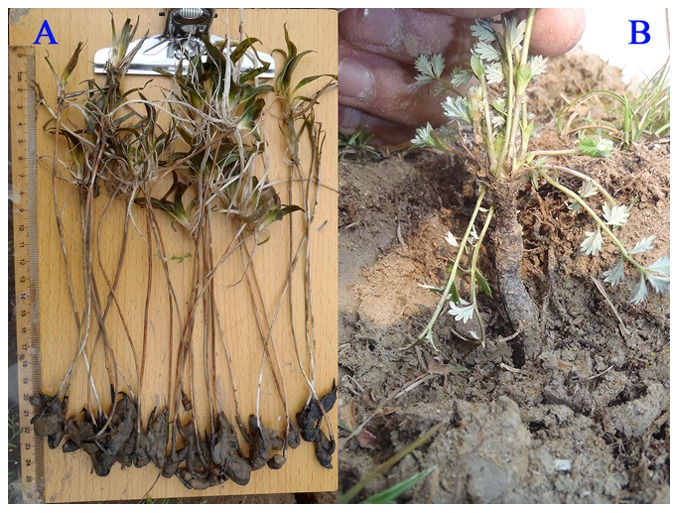
Plants of vegetation survey. A: *Vallisneria spiralis* and B: *Potentilla limprichtii*.

Conservation of Poyang Lake has become a national and international focus over the past three decades. PLNNR was established in 1988, and was designated as a Ramsar site in 1992. The PLNNR covers an area of 224 km^2^ in the northwest corner of the lake, where the Xiu and Gan Rivers join Poyang Lake. There are three types of habitat for waterbirds, namely shallow water, mudflat and wet meadow. In autumn, when water level drops, vast mudflats, shallow water areas, and grasslands are exposed; and nine shallow lakes (Figure 2) emerge comprising the primary water birds habitats within the Reserve. At shallow water and mudflat habitats, submerged plants, in particular *V. spiralis*, are exposed for the cranes. At a slightly higher elevation (often less than 1 meter higher), vegetation develops into wet meadow, where *Potentilla limprichtii* (Figure 3) is abundant.

### Siberian crane population census at different habitats

We conducted Siberian crane population census at different habitats (i.e. wet meadow, mudflat and shallow water area) in 2011 winter (24th February to 25th February) in all the nine lakes of PLNNR. We employed a point count method for all lakes using scoping scope (Swarovski ATS 80 HD 20–60×80). In smaller lakes (i.e. Shahu, Changhuchi, Zhonghuchi, Meixihu, Xianghu and Zhushihu), we counted birds at a fixed high vantage point. The area of smaller lakes ranges from 2–14 km^2^, from the observation points; we have a full view of the lakes. In other bigger lakes, we selected two or three points to count Siberian crane depending on the size of the lake to ensure that the entire lake was visible and surveyed.

### Food resource surveyed

We investigated crane's food sources and availability by randomly placing a number of 1 m×1 m quadrats within the three habitat types, where cranes foraged in the early winter. Sediment to the depth of 30 cm within the quadrat was excavated, and tubers and/or roots were gathered for identification and counting. As the length of male crane's bill is about 18.8 cm [19], all reachable food was included. We sampled two lakes, Banghu and Meixihu for Siberian crane food source. Banghu Lake is the main foraging ground of the Siberian crane, and during the whole wintering season, there were always more than 2,000 cranes presented. In Meixihu Lake, we counted over 600 cranes in December 2010. In total, we collected 25 and 6 sediment samples for estimating tuber density at Banghu Lake and Meixihu Lake, respectively. We also collected 38 and 10 samples in Dahuchi Lake and Banghu Lake, respectively. The two lakes were the main locations where we observed cranes foraging on grassland.

We also conducted surveys for *V. spiralis* in Banghu, Sahu, Dahuchi and Changhuchi during the period of May 3–6, 2011 and May 9–12, 2012 after the migrating waterbird backing to their breeding grounds. Plant buds and newly germinated plants within randomly placed quadrats (1 m×1 m) were counted and recorded to compare food abundance in the winters of 2011 and 2012.

### Crane behavior observation

We conducted Siberian crane behavioral observations along transects at three distinct habitats from the 9th to 17th of March, 2011. We did not conduct field observation in rainy, snowy or strong windy days to avoid biased measurements. All transects were located within the PLNNR (Figure 1); and transects were not repeated to avoid sampling the same individual. Behavioral observations were conducted during daylight hours (i.e. from sun rise to sunset). We examined individual bird behavior using focal sampling technique [30]. Focal sampling is an instantaneous measure of individual bird behavior, and provides an overall estimate of proportions of an individual engaged in different behaviors. Behaviors were classified into 1 of 6 categories (modified from Davis and Smith 1998) [31]: foraging (all those behaviors involving food capture attempts, food item handling, and consumption such as stabbing, pecking, nibbling, tugging, thrashing, bill-flicking, and bill-wiping), locomotion (all ambulatory activities included walking, running, hopping, and leaping), alerting (stationary with neck upright, surveying surroundings), maintaining (bathing, preening, or drinking), resting (standing by two legs or single leg, or bedding down), and other behaviors (chasing, pecking, biting, flight and courtship).

During an observation session, one randomly selected crane was followed visually for 20 consecutive minutes using a scoping scope. The observed crane was identified as either juvenile or adult by morphological features to investigate if the cranes display different behavior pattern at different development stages. Behaviors were recorded into a voice-activated tape-recorder, transcribed, and converted to the percentage of the crane engaged in each behavior.

### Data analysis

Proportion of all cranes engaged in foraging, locomotion, alert, maintenance, resting and other activities was calculated separately for each observation session, for birds using each habitat type at either juvenile or adult development stages. For each observation session, we also calculated the activity changing frequency. We used permutational (formerly known as nonparametric) multivariate analysis of variance (MANOVA) to test for differences in overall behavior displayed at different habitats (wet meadows, mudflats and shallow water area) for juvenile and adult birds. We chose permutational MANOVA to analyze behavior pattern primarily based on the following two considerations:

- the dependent variable categories were not independent of each other (percent of time engaged in one behavior influenced the percent of observed in other behaviors). Multivariate analysis considers correlation among multiple dependent variables, which individual analysis of variances (ANOVA) do not, and is usually more powerful than a series of separate ANOVAs [32].
- the time activity data are not normally distributed, even with arcsine-square-root- and log- transformation (p<0.001). Although the factorial MANOVA has less restrictive assumptions regarding normality of the data and equality of covariance matrices than repeated measures analysis of variance [33], its nonparametric alternative is more appropriate.

Following significant permutational MANOVA test, which indicated that the cranes displayed significantly different behavior pattern in different habitats/development stages, we used the univariate Kruskal-Wallis test (the nonparametric equivalence of univariate ANOVA) to examine differences in individual activity among habitat types and development stages and post hoc pairwise comparisons (Mann-Whitney test) were used to investigate significant results. We used Mann-Whitney test because of the relatively small sample size for each habitat by development stage. The densities of the bud and plantlet of *V. spiralis* in different lakes were also assessed with Kruskal-Wallis test. All statistical tests were performed with R 2.15.0 [34].

## Results

### Siberian crane population and its spatial distribution in PLNNR

A total 2,465 cranes were recorded in 4 out of 9 the lakes of the PLNNR in 2011 winter (24th and 25th February), which is more than 70% of its global population. The proportion of the cranes observed feeding at shallow water, mudflat and wet meadow accounts for 48%, 7% and 45% respectively (Table 1).

**Table 1:** Siberian crane abundance observed at different habitats in different lakes.

Habitat	Dahuchi lake	Banghu lake	Xianghu lake	Dachahu lake	Total
Shallow water	0	1,200	0	0	1,200
Mudflat	38	125	0	16	179
Wet meadow	66	984	8	28	1,086
Total	104	2,309	8	44	2,465

### Food plant density and its spatial distribution

We collected 31 *V. spiralis* samples. The average density of tuber was 1.24 ind/m^2^ (individuals per square meter) and 0.17 ind/m^2^ in Banghu Lake and Meixihu Lake, respectively. The average density of the main root of *Potentilla limprichtii* J. in Banghu Lake and Dahuchi Lake was 182.0 ind/m^2^ and 125.5 ind/m^2^ (Table 2).

**Table 2:** The density of the tubers and roots (mean±SD) in different lakes.

Species	Banghu lake	Meixihu lake	Dahuchi lake
*Vallisneria spiralis* Tubers(ind/m^2^)	1.24±1.61	0.167±0.37	
*Potentilla limprichtii* Roots(ind/m^2^)	182.0±167.0		125.5±33.8

We collected a total of 795 samples on bud and plantlet of *V. spiralis*. The survey results showed that the densities of the plantlets were significantly lower in the 2011 than in the 2012 for all of 4 lakes (chi-squared = 51.682, p<0.001, Table 3). In particular, the density of plantlet in 2012 was 42.6 times more than that in 2011 in Dahuchi Lake.

**Table 3:** The density (ind/m^2^) (mean±SD) of the *V. spiralis* buds and the plantlets in different lakes between 2011 and 2012.

	Banghu Lake		Shahu Lake		Changhuchi Lake		Dahuchi Lake		Average	
year	2012	2011	2012	2011	2012	2011	2012	2011	2012	2011
Density	59.5±39.1	24±13.9	15.8±19.7	14.7±5.9	59.7±47.3	25.6±10.8	17.1±19.3	0.4±0.8	38.6±38.8	18.2±13.6
sampling size	160	147	80	102	40	90	110	66	390	315
chi-squared	104.437***		5.593*		19.721***		48.164***		51.682***	

*** p<0.001;

** p<0.01;

* p<0.05.

### Behavioral of crane at different habitat

We collected time activity data for 178 individual cranes from 9^th^ to 17^th^ March 2011, representing a total of 3,545.6 minutes of observation (Table 4). The pooled data shows that the cranes spent the most time for foraging (76%) and the least time being alert (2%). Other activities such as resting, locomotion, maintenance, and the rest including courtship and fighting were 8%, 6%, 5%, and 2%, respectively (Table 4).

**Table 4:** Summary of the collected time budget data (mean) of Siberian crane in different habitats by development stages.

Habitat	Stage	Sample size	Foraging	Alert	Locomotion	Resting	Maintenance	Others	Change Frequency
Shallow lakes	Adult	52	76%	0%	5%	8%	8%	4%	12.8
	Juvenile	26	88%	0%	3%	8%	1%	0%	8.1
Mudflat	Adult	11	89%	0%	1%	3%	7%	0%	9.2
	Juvenile	12	84%	0%	10%	0%	0%	4%	8
Wet meadow	Adult	53	64%	7%	9%	13%	5%	3%	29.2
	Juvenile	24	84%	1%	6%	6%	3%	1%	17.2
Total		178	76%	2%	6%	8%	5%	2%	17

#### Overall behavior pattern

The nonparametric MANOVA test (Table 4) revealed that a significant multivariate main effect for both habitat type (*F*_2, 175_ = 3.995, *p* = 0.008) and development stage *F*_1, 176_ = 8.163, *p* = 0.001), but not their interaction (*F*_2, 175_ = 1.612, *p* = 0.164). The standardized canonical coefficients indicated that the Juvenile spent more time for foraging and moving around and less time alerting (Table 4). Among the three habitat types, the cranes spent less time for foraging and the most time alerting in wet meadows and inconsistent for other behaviors (Table 5).

**Table 5:** Nonparametric MANOVA of development stage and habitat type on Siberian crane activity time.

Factor	df	F	P	Coefficients						Significant differences
				Forage	Alert	Locomotion	Rest	Maintenance	other	
Stage	1, 177	7.76	0.001**	4.65	−0.93	0.59	−1.53	−2.57	−0.21	Juvenile vs. Adult
Habitat	2, 176	3.76	0.008**	1.11	−1.25	−1.76	1.63	0.34	−0.06	Water vs. Meadow
				5.93	−1.24	0.12	−4.75	−0.24	0.19	Mudflat vs. Meadow
Stage: Habitat	2, 175	1.612	0.164n.s							

*** p<0.001;

** p<0.01;

* p<0.05; and

n.s = not significant (p>0.05).

Stage refers to development stage (juvenile or adult). The coefficients are the standardized canonical correlation coefficient for individual activities.

#### Effects on individual activities

The following univariate nonparametric Kruskal-Wallis tests showed that the effects were inconsistent for individual activities (Table 6). Time foraging, time alerting and activity changing frequency were among the most affected variables (p<0.001), while time other activities was insensitive to both habitat type and development stage (p>0.05, Table 6).

**Table 6:** Kruskal-Wallis test of the effects of development stage and habitat type on Siberian crane activity time.

Factor	Forage		Alert		Locomotion		Rest		Maintenance		other		Frequency	
	X^2^	p	X^2^	p	X^2^	p	X^2^	p	X^2^	p	X^2^	p	X^2^	p
Stage	12.45	0.00***	12.637	0.00***	4.12	0.04*	6.51	0.01**	3.66	0.06n.s	3.14	0.08n.s	14.56	0.00***
Habitat	25.96	0.00***	72.512	0.00***	13.1	0.00**	10.71	0.00**	7.38	0.02*	5.73	0.06n.s	53.58	0.00***

*** p<0.001;

** p<0.01;

* p<0.05; and

n.s = not significant (p>0.05);

#### Foraging time at different habitat

The two-sample comparison was focused on the most sensitive variables (i.e. time foraging, time alerting and change frequency, Table 6). In addition, the variables were separated by age because of the significant difference (chi-squared = 12.45, p<0.001, Table 6).

**Figure 4:**
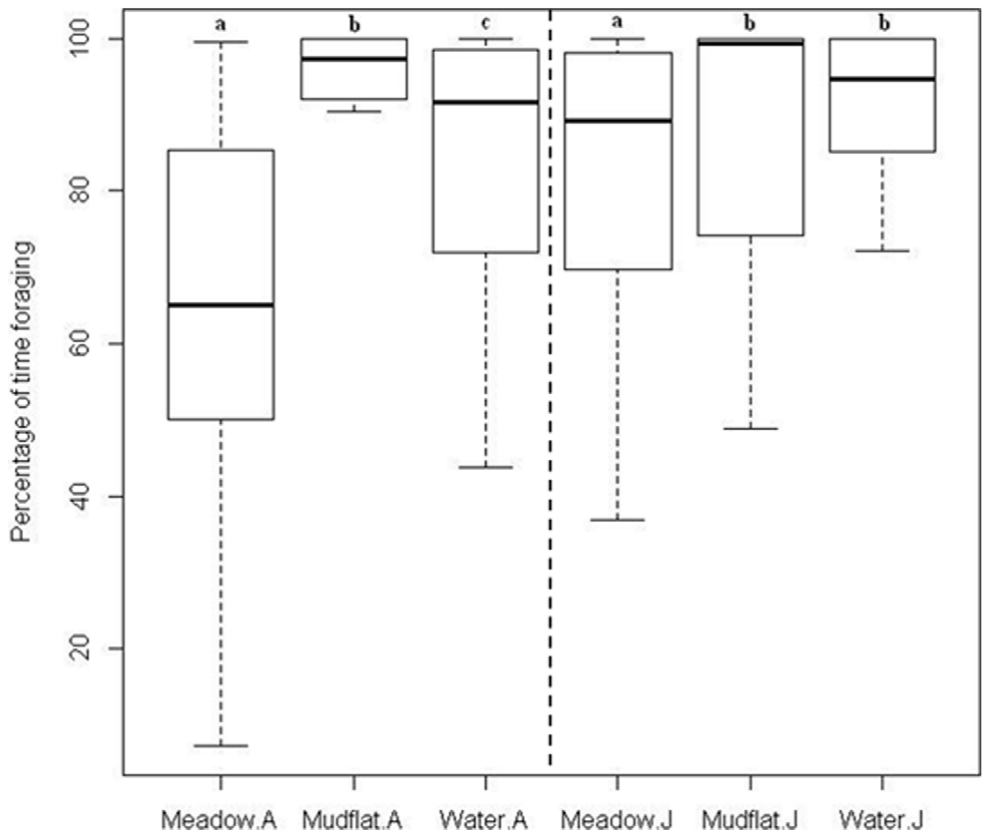
Differences in percentage of time foraging. In a 20-min observation session for the Siberian crane, the differences in percentage of foraging time at three habitat types in the Poyang Lake National Nature Reserve grouped by development stages (A = Adult and J = Juvenile). Within the same stage group, habitats with the same lowercase letter did not differ significantly (p>0.05) based on Mann-Whitney test.

The Adult Siberian crane spent most of their time for foraging in all habitats (median time foraging is 97.25%, 91.63% and 65.08% for mudflat, shallow water and wet meadow, respectively, Figure 4). The Mann-Whitney testing confirmed that the crane spent significantly less for foraging at wet meadow than at mudflat and shallow water (Figure 4). In addition, the difference between shallow water and mudflat is also significant.

For the Juvenile crane, the variation in foraging time between different habitat types is similar to that of adult crane (99.21%, 94.71% and 89.16% for mudflat, shallow water and wet meadow, respectively); and the percentage of time foraging is significantly lower at wet meadow than at shallow water and mudflat, whereas, no significant difference was observed between shallow water and mudflats (Figure 4).

**Figure 5:**
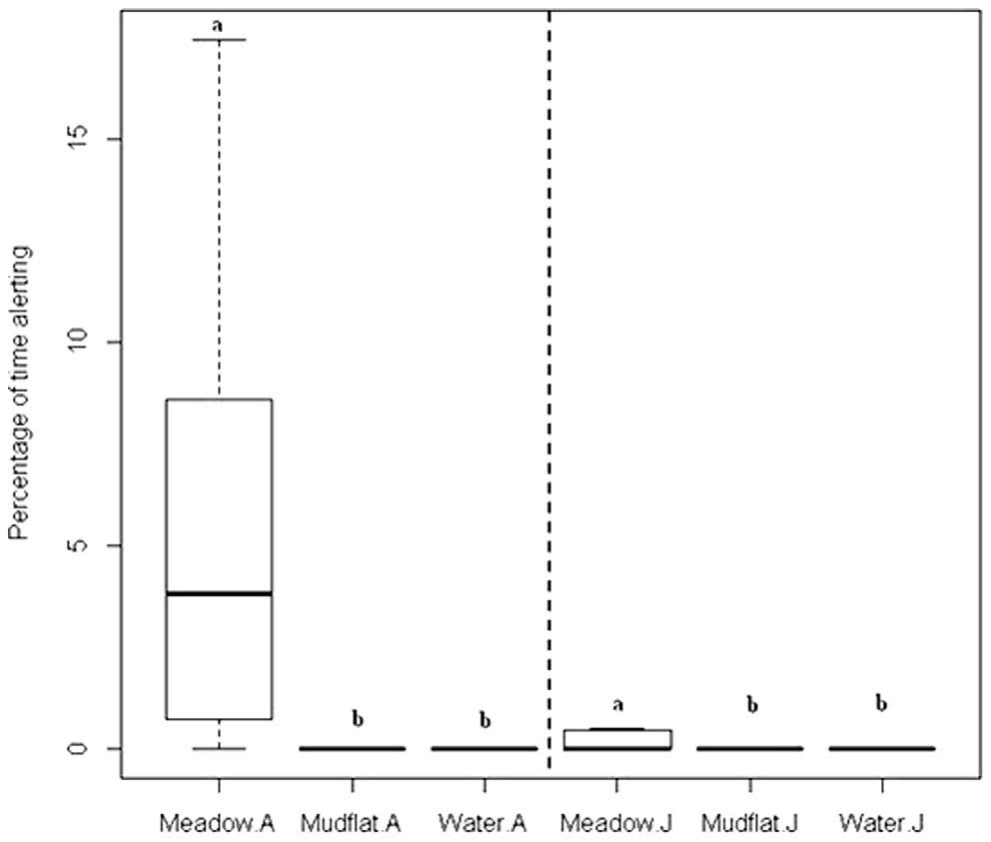
Differences in percentage of Alerting time. In a 20-min observation session for the Siberian crane, the differences in percentage of alerting time at three habitat types in the Poyang Lake National Nature Reserve grouped by development stages (A = Adult and J = Juvenile). Within the same stage group, habitats with the same lowercase letter did not differ significantly (p>0.05) based on Mann-Whitney test.

The cranes spent only a very small fraction of their time being alert; and in most cases, the median value is zero (Figure 5). Although two-sample comparing Mann-Whitney tests were conducted as for time foraging, we can only report that the time alerting is significant higher than zero at wet meadows due to the many zero values for mudflat and shallow water.

#### Behavior changing frequency at different habitat

**Figure 6:**
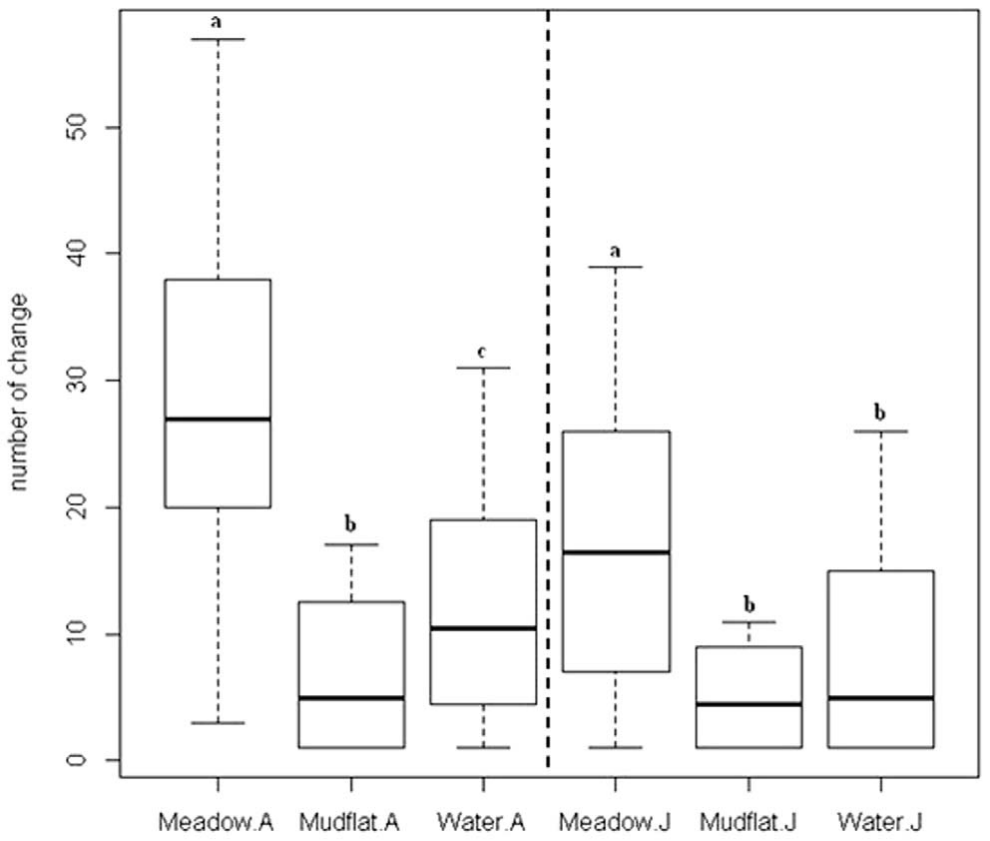
Differences in behavior changing frequency. In a 20-min observation session for the Siberian crane, the differences in behavior changing frequency at three habitat types in the Poyang Lake National Nature Reserve grouped by development stages (A = Adult and J = Juvenile). Within the same stage group, habitats with the same lowercase letter did not differ significantly (p>0.05) based on Mann-Whitney test.

The adult crane changed behavior more frequently at wet meadow, followed by at shallow water and at mudflat (median equals to 29.2, 12.8, and 9.2 times per 20 minutes for wet meadow, shallow water and mudflat, respectively, Figure 6).

As with time foraging, the activity change frequency for the juvenile crane followed that of the adult, although the absolute value was lower (Figure 6). However, the statistic test revealed that the juvenile crane did not differ significantly in terms of activity change frequency at shallow water and mudflat (Figure 6).

## Discussion

### Why Siberian cranes selected wet meadows over shallow waters and mudflats?

This paper is probably the first time to document the phenomenon of large flock of Siberian cranes foraging on grasslands and shifting their diet. Animal habitat selection and use can be influenced by a range of factors including food availability, competition pressure, and predation risk, reproductive and social behaviors [1], [2], [40], [41], [42].

Lack of food might be the main driver for shifting habitat in this occasion. In the spring of 2010, the water level at Xingzi hydrologic Station (XZHS) was higher than the long-term mean. The elevated water level reduced the underwater light intensity, which in turn might reduce the growth and tuber yield of *V. spiralis* [35], which is the main food of the wintering Siberian cranes. *V. spiralis* is the predominant aquatic species in the shallow waters of the Poyang Lake [36] and often propagates by means of tuber clonal reproduction which can lead to dense stands. The tuber and seeds have been observed germinating from March to April, producing tuber from May–August, and flowering and fructify from August to October [37]. The vegetation investigation results showed that the density of *V. spiralis* tubers was lower in the 2010–2011 wintering season, compared to other years (Table 7) [38]. The water level at XZHS was higher than 17 m above sea level for more than 100 days in the 1998 and 2010. The long-lasting high water level during the growing season might reduce the yield of tuber.

**Table 7:** The density of *vallisineria spiralis* tuber (ind/m^2^) in different year.

Year	Banghu lake	Dahuchi lake	Water level (m)^A^	Days^B^
1998.12 [43]	15.8		22.52	111
2005.11 [44]	179±75.5	41±28.33	19.05	53
2010.10 [38]		0.20	20.28	105
2011.5	24	0.40	20.28	105
2012.5	59.5	17.10	17.42	8

A : The highest water level in summer at XZHS;

B : Days of water level ≥17 m in previous growing season.

In China, Siberian cranes feed primarily on stems and tubers of *V. spiralis*, but also on pondweed (*Potamogeton malainus*) and small freshwater clams [39]. In the alternative environment with different food, the Siberian crane might adjust its foraging behavior. For instance, at shallow waters and mudflats, the cranes are mainly engaged in probing the mud for food while they commit most of the time for identifying targets and digging at wet meadow.

### Will Siberian crane remains foraging on the wet meadow even if there is enough food in the shallow water and mudflat?

The permutational MANOVA confirmed our second hypothesis that the Siberian crane displayed distinct behavior patterns at the alternative habitat for both the juvenile and adult. The most notable difference was found in time allocation for foraging, alerting, as well as frequency of behavioral change. The crane spent significantly less time for foraging, more time for alerting, and the frequencies change from one activity to another increased significantly(p<0.001, table 6) at wet meadow (Table 4). Such behavior changes might indicate that the cranes did not feel secured when foraging on a new environment.

The nonparametric Kruskal-Wallis and Mann-Whitney tests supported our third hypothesis that the Siberian crane spent more time in vigilant activities (Figure 5). In Poyang Lake, with the habitats of concentric shape, the areas with shallow water provide less disturbance and adequate distance from human activities. In wet meadow, however, we found that the Siberian cranes had to share the habitat with other waterfowl such as white-naped crane (*G. vipio*), hooded crane (*G. monacha*) and common crane (*G. grus*). The added disturbances from other waterfowl increased the proportional time for alerting. Furthermore, the usual habitat of shallow waters and mudflats provide an ideal open environment for the Siberian crane with less disturbance from human and other animals. Once stepped out of its customary zone, the Siberian cranes feel uneasy and change behavior more frequently. Giving enough food resources in the shallow waters, e.g., tubers of *V. spiralis*, the Siberian cranes might not select and use wet meadows, and that may be the reason why they are observed foraging at shallow waters and mudflats most of the time. Our survey in the wintering season of 2011 confirmed this. In 2011, there were abundant *V. spiralis* with an average density of 18.2±13.6 (Table 3). Siberian cranes were observed foraging in the shallow water during the whole winter, and we did not find a single crane on grasslands.

### The importance of wet meadows for the survival of Siberian cranes

*V. spiralis* is the predominant aquatic species in the shallow waters of the Poyang Lake [36], its biomass is normally enough sustain Siberian crane and other water birds such as tundra swan (*Cygnus columbianus*) and swan goose (*Anser cygnoides*). In PLNNR, tubers have abundant reserves and distribute in cluster of patches, which provide ideal food resources for Siberian crane. *P. limprichtii* is the main hygrophyte species at wet meadow. It is distributed on the edge of the *Carex* meadow, which is the main wetland vegetation in Poyang Lake. *P. limprichtii* is a perennial herb, which has stout roots, and flowering and fruiting from November to March. Therefore, cranes can easily indentify the plant with flowers, and dig out the roots for food.

For a long time, Siberian crane has been considered as the most endangered cranes, and is threatened by the development of the Three Gorges Dam, as well as the numerous dams on the Yangtze River and its tributaries [10]. Jiangxi Province planned to build a dam to control the hydrological variations in Poyang Lake. The dam was designed to directly control the Lake's outlet (to the Yangtze), thus the Lake water level. These dams will dramatically change the hydrological regime of Poyang Lake, which could have adverse impacts on the growth of *V. spiralis*. Underwater light condition in bud germinating season is one of the key environmental factors for the survival and growth of this species [36]. Therefore, the enhanced lake water level, such as in 1998 and 2010, could deteriorate the light density reaching the canopy of *v. spiralis*, causing food shortage for Siberian cranes. Our results showed that when food was limited, Siberian cranes, although with highly specialized morphology adapting to shallow water habitat, had the flexibility to shift their diet and to acclimatize to wet meadow for survival, at least temporarily.

## Conclusion

Under extreme conditions, the Siberian cranes can find alternative habitat and have their diet shift from *V. spiralis* to *P. limprichtii*. When Siberian cranes shift to a new environment, they displayed distinct behavior patterns by allocating significantly less time foraging, but spending more time alerting, and frequently changing their behavioral activities. When the conditions improved, it will return to its optimal foraging habitat.
